# Analytic Computation of Vibrational Circular Dichroism Spectra Using Second-Order Møller–Plesset Perturbation Theory

**DOI:** 10.1021/acs.jctc.5c00047

**Published:** 2025-03-25

**Authors:** Brendan M. Shumberger, Kirk C. Pearce, T. Daniel Crawford

**Affiliations:** Department of Chemistry, Virginia Tech, Blacksburg, Virginia 24061, United States

## Abstract

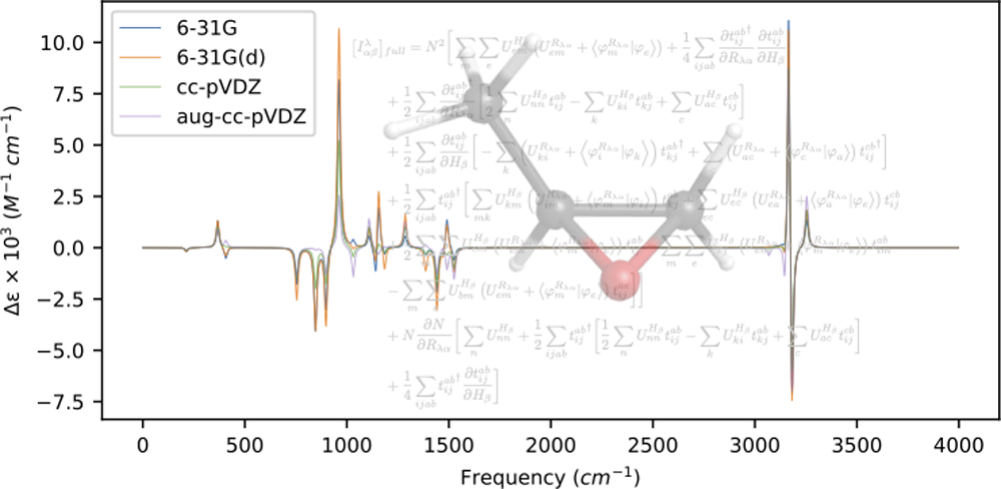

We present the first analytic-derivative-based formulation of vibrational circular dichroism (VCD) atomic axial tensors for second-order Mo̷ller–Plesset (MP2) perturbation theory. We compare our implementation to our recently reported finite-difference approach and find close agreement, thus validating the new formulation. The new approach is dramatically less computationally expensive than the numerical derivative method with an overall computational scaling of . In addition, we report the first fully analytic VCD spectrum for (*S*)-methyloxirane at the MP2 level of theory.

## 1 Introduction

From its advent, vibrational circular dichroism (VCD) — the differential absorption of left- and right-circularly polarized infrared light by a chiral compound — has been a challenge to quantum chemistry because the requisite rotatory strengths vanish within the Born–Oppenheimer approximation. The VCD rotatory strength is obtained from the dot product of the electric- and magnetic-dipole transition-moment vectors between vibrational states, and while the former may be straightforwardly computed via differentiation of the expectation value of the electric dipole moment operator in the electronic ground state, the same approach fails for the latter because the corresponding expectation value is zero for closed-shell states. In the 1970s and 1980s, a number of ad hoc models were put forward in an attempt to simulate VCD spectra,^1−10^ but it was Stephens’s 1985 formulation^11^ of the magnetic-dipole transition moment [commonly referred to as the atomic axial tensor (AAT)] that provided the first, general ground-state VCD approach, requiring only the overlap of wave function derivatives with respect to nuclear coordinates and the external magnetic-field.

The first computations of VCD AATs using Stephens’s approach were reported at the Hartree–Fock (HF) level by Lowe, Segal, and Stephens in 1986 using numerical differentiation of the ground-state wave function.^12,13^ While this approach provided useful insights and benchmarks, it was not sufficient for practical calculations due to the need for complex wave function representations (and algebra) for finite magnetic-field perturbations. In 1987, Amos et al.^14^ described the first implementation of analytic-derivative techniques for the calculation of HF-level AATs, requiring solution of the first-order coupled-perturbed Hartree–Fock (CPHF) equations for the derivatives of the molecular orbital (MO) coefficients, as well as half-derivative overlap integrals. Six years later, Bak et al. reported their extension of Stephens’s AAT formulation to the multiconfigurational self-consistent field (MCSCF) level of theory^15^ allowing for the inclusion of static electron correlation effects while simultaneously tackling the origin independence problem by introducing gauge-including atomic orbitals (GIAOs).^16,17^ Soon thereafter, Stephens and co-workers^18^ simulated VCD spectra using second-order Mo̷ller-Plesset (MP2) theory and density functional theory (DFT) though only the harmonic force fields and the electronic-dipole transition moments [atomic polar tensors (APT)] were computed using these methods; the AAT was computed only at the HF level of theory. Building upon this work, in 1996 Cheeseman et al.^19^ carried out the first full simulation of VCD spectra using DFT, including the use of GIAOs.

Recently, we reported the first simulations of VCD spectra at the MP2 and configuration interaction doubles (CID) levels of theory.^20^ Similar to Stephens’s approach in the mid-1980s using HF theory, we carried out the calculation of the AATs using numerical differentiation of the corresponding wave functions. However, the required determinantal expansions lead to much greater complexity and computational cost in the MP2/CID cases compared to HF due to the need to evaluate overlaps between doubly excited determinants in nonorthonormal bra and ket bases. This yields an  scaling of the method, thus limiting our analysis to only small molecules with modest basis sets.

Here, we present the first analytic-derivative approach to computing MP2 AATs. Our implementation eliminates the need for complex arithmetic and nonorthonormal MOs, yielding an overall scaling of . In the next section, we will outline our derivation of the working equations, followed by validation of the results by comparison between finite-difference and analytic AATs. We then report the first MP2VCD spectra simulations for (*S*)-methyloxirane for several basis sets.

## 2 Theory

In Stephens’s formulation of VCD rotatory strengths,^11^ the electronic contribution to the AAT, , is obtained from the overlap between derivatives of the ground state wave function, Ψ_G_, with respect to nuclear displacement, *R*_λα_, and the external magnetic field, *H*_β_,
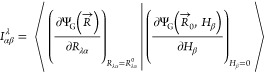
1which is evaluated at the equilibrium geometry and at zero field. The indices λ and α denote the nucleus and Cartesian axis of the λ^th^ nucleus’ displacement, respectively, while β denotes the Cartesian axis of the magnetic field. By approximating the wave function using first-order Mo̷ller-Plesset theory,
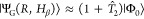
2we can obtain the second-order Mo̷ller-Plesset (MP2) AAT where the *T̂*_2_ operator is
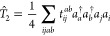
3and Φ_0_ is the HF reference determinant. We use the standard indexing scheme where *i*, *j*, *k*, ... denote occupied spin–orbitals, and *a*, *b*, *c*, ... denote virtual spin–orbitals. Indices *p* and *q* will denote MOs appearing in either subspace. Inserting eqs 2 and 3 into eq 1 with subsequent application of the second quantized operators onto the HF reference determinant yields the intermediately normalized AAT (denoted by the subscript int) of the form
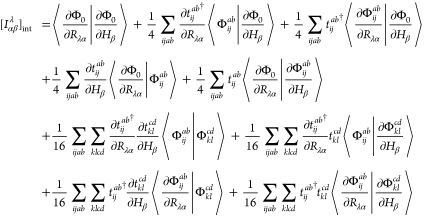
4where  is a doubly substituted determinant. Evaluation of the overlap between wave function derivatives can be performed by considering the derivative of an MO, φ_*p*_,
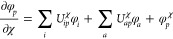
5In eq 5,  is a CPHF coefficient and  is the derivative of an atomic orbital (AO) with respect to some arbitrary perturbation χ, transformed into the MO basis (also known as a core derivative). Solutions to the CPHF equations for nuclear displacements and magnetic field perturbations have been reviewed by Yamaguchi et al.^21^ and by Amos et al.,^14^ among others, and we direct readers to these works for further information. Utilizing the derivative product rule on the Slater determinant,
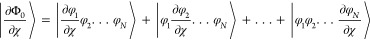
6and inserting eq 5, we can define the derivative of our reference wave function in a second-quantized notation
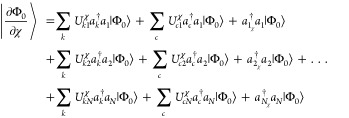
7where  is a creation operator for the core derivative of φ_*p*_ with respect to χ. (Note that the derivatives of the spin–orbitals are not orthogonal to their unperturbed counterparts.) This expression can be reduced to
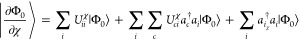
8Similar to the derivative of the reference determinant, we can express the derivative of the doubly excited determinant in a second quantized notation as,
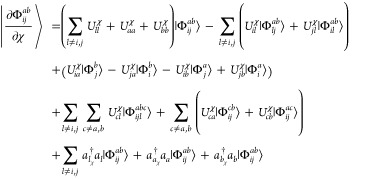
9where we have separated operator strings by their action on the reference determinant and removed redundant operator pairs. With our derivative determinants in eqs 8 and 9, we can write the explicit forms for the bra-state derivatives with respect to nuclear displacements and ket-states derivatives with respect to magnetic-field perturbations. For the HF reference determinant, we obtain
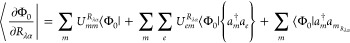
10and
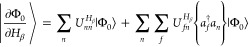
11Note that terms involving core derivatives do not appear in eq 11 because we are not including field-dependent basis functions (GIAOs) in the current formulation. For the corresponding derivatives of the doubly excited determinants, including the wave function amplitudes and associated spin–orbital summations allows us to take advantage of the symmetry of the indices yielding the simplified expression,
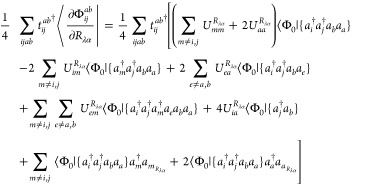
12and
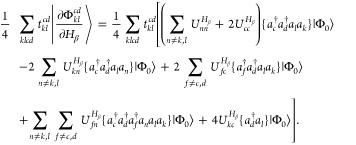
13The overlaps between eqs 10–13 may be evaluated with the standard Wick’s theorem contraction rules,^22^ as well as additional rules for the core derivatives, viz.,
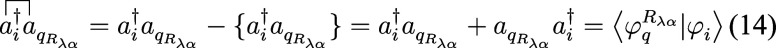
14
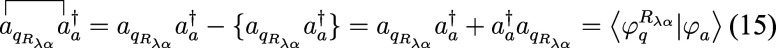
15
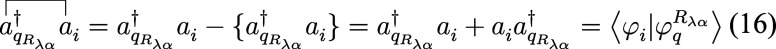
16and
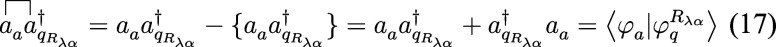
17where the terms on the far right-hand side of each equation are half-derivative overlap integrals. With these equations in hand, we may now derive the various contributions to eq 4 using Wick’s theorem and retaining only the fully contracted terms. The first term is the HF contribution, which evaluates to
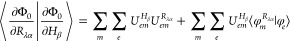
18where we have used the relationship
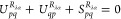
19which is obtained from the derivative of the MO orthonormality condition. The second and fourth terms in eq 4 include the derivative of the reference determinant projected onto the doubly excited determinant. Since the derivative of the reference determinant includes at most single excitations [cf. eqs 10 and 11], no full contractions can be produced from these terms. Thus,
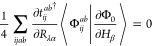
20and
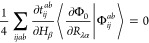
21We find that the third term,
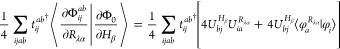
22and fifth term,
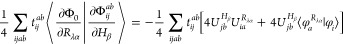
23in eq 4 exactly cancel. As such, only the HF term provides any contribution to the AAT from among the first five terms. The sixth term includes only derivatives of *T̂*-amplitudes and thus simplifies to
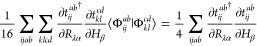
24The seventh and eighth terms in eq 4 are
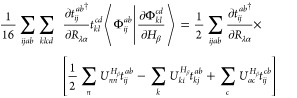
25and
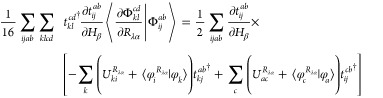
26respectively. The final term in eq 4 involves the overlap between doubly excited derivative determinants and, as a result, is the most complicated in terms of Wick’s theorem contractions. The resulting expression is
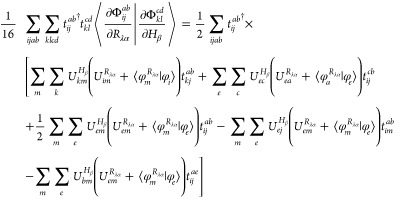
27where we note that  is symmetric and the unperturbed *T̂*-amplitudes are real which leads to the self-cancellation of several terms involved in the derivation.

The combination of eqs 18 and 24–27 results in the analytic spin–orbital expression for the AAT assuming intermediate normalization of the ground-state wave function. However, Stephens’s formulation for the AAT assumes fully normalized wave functions, which we thus include in our first-order MP wave function as,
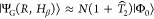
28Differentiation of this wave function leads to
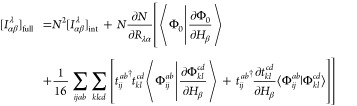
29for which  includes only terms eqs 18, 24–27. Additionally, differentiation of the normalization factor, *N*, yields
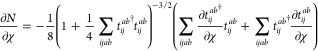
30This derivative is zero for magnetic-field perturbations because the derivative *T̂*-amplitudes are pure imaginary quantities, and thus the final two terms in parentheses on the right-hand side exactly cancel. The AAT with fully normalized wave functions is then given as
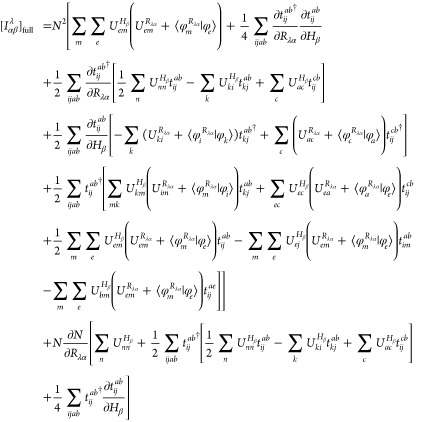
31

## 3 Computational Details

We have implemented the analytic-gradient scheme for computing the AAT described above in the open-source Python package apyib.^23^ To validate our implementation, we compared the fully normalized AATs with those produced by MagPy which computes MP2 AATs using a finite-difference procedure.^24^ Both of these codes use the Psi4 quantum chemistry package to provide the necessary integrals.^25^ We should note that, in order to compare finite-difference and analytic formulations, we must assume that the perturbed HF orbitals are canonical, and thus we include *U*_*pq*_^χ^ CPHF coefficients corresponding to both dependent and independent pairs. We will consider the use of noncanonical perturbed MOs to simplify the computations in future work.^26^

We carried out comparisons between the analytic and finite-difference AATs using several small molecular test cases, including the hydrogen molecule dimer, water, and (*P*)-hydrogen peroxide with multiple basis sets. However, we have chosen (*P*)-hydrogen peroxide in conjunction with the 6–31G basis as a representative example to demonstrate the correspondence between the two approaches. For the finite difference procedure, geometric displacements and magnetic field perturbations were set to 10^–4^ a.u. Additionally, we converged SCF energies to 10^–13^ a.u. for both analytic- and numerical-derivative calculations.

Furthermore, we carried out computations of rotatory strengths and corresponding VCD spectra for (*S*)-methyloxirane with the 6–31G, 6–31G(d), cc-pVDZ, and aug-cc-pVDZ basis sets^16,27−32^ using both a common geometry/common Hessian scheme and one where the geometries and Hessians were optimized and computed at the same level as the rotatory strengths. The CFOUR quantum chemistry program was used for optimizing geometries as well as for computing the Hessian and APTs.^33^ All calculations for (*S*)-methyloxirane were converged to 10^–10^ a.u. for the energy and 10^–8^ a.u. for the gradients. Cartesian geometries for all data reported in this work are available in the Supporting Information (SI). All electrons were correlated in all calculations reported here.

## 4 Results and Discussion

### 4.1 Comparison between Analytic and Numerical Differentiation

The AAT obtained for (*P*)-hydrogen peroxide using the analytic-gradient method discussed above agrees closely with that obtained using the finite-difference procedure, and can be observed from the data in Table 1. The largest discrepancies in individual AAT elements between the two procedures are on the order of 10^–7^ a.u., with most around 10^–9^. This result is not unexpected as we observe similar differences between the AATs computed at the HF level of theory using these two approaches. We believe this provides strong support for the correctness of both our working equations and our implementation.

**Table 1:** Electronic MP2 AATs (a.u.) for (*P*)-Hydrogen Peroxide Computed with the 6-31G Basis Using Analytic-Gradient Methods and Finite-Difference Procedures

	analytic			finite difference		
	*B*_*x*_	*B*_*y*_	*B*_*z*_	*B*_*x*_	*B*_*y*_	*B*_*z*_
H_1*x*_	0.0060845318	0.0243783889	0.0065127385	0.0060845320	0.0243783887	0.0065127390
H_1*y*_	0.0385652752	–0.1544999813	0.3151731127	0.0385652752	–0.1544999797	0.3151731091
H_1*z*_	–0.0693503855	–0.1656548215	0.1578557162	–0.0693503859	–0.1656548213	0.1578557139
H_2*x*_	0.0060845318	0.0243783889	–0.0065127385	0.0060845319	0.0243783886	–0.0065127383
H_2*y*_	0.0385652752	–0.1544999813	–0.3151731127	0.0385652752	–0.1544999799	–0.3151731086
H_2*z*_	0.0693503855	0.1656548215	0.1578557162	0.0693503859	0.1656548214	0.1578557132
O_3*x*_	–0.0136643683	0.1131764309	–0.1508660802	–0.0136643651	0.1131764020	–0.1508660412
O_3*y*_	–0.0449758129	–0.0553557340	1.1585427903	–0.0449758045	–0.0553557163	1.1585425174
O_3*z*_	0.1014871485	–1.0889005631	0.0597385980	0.1014871294	–1.0889003119	0.0597385786
O_4*x*_	–0.0136643683	0.1131764309	0.1508660802	–0.0136643651	0.1131764030	0.1508660409
O_4*y*_	–0.0449758129	–0.0553557340	–1.1585427903	–0.0449758045	–0.0553557172	–1.1585425196
O_4*z*_	–0.1014871485	1.0889005631	0.0597385980	–0.1014871295	1.0889003110	0.0597385803

As noted by Amos et al.,^14^ the advantage of the analytic approach over numerical differentiation in computing the AATs is already substantial even at the HF level of theory. In their work, they observed that the analytic formulation for HF AATs required only approximately two to three times more computational effort than a single SCF calculation, whereas the numerical approach requires multiple SCF calculations — (3*M* + 3) × 2, where *M* is the number of atoms — as well as the need for complex arithmetic.

The differences between the numerical- and analytic-derivative approaches are exacerbated at the MP2 level. For the finite-difference procedure, evaluation of contributions involving the overlap between derivatives of doubly excited determinants in the bra and the ket such as the last term on the right-hand side of eq 4 require computations of the overlap between doubly excited determinants represented in different MO basis sets due to displacements of δ*R*_λα_ for the bra wave function and of δ*H*_β_ for the ket wave function. For each combination of doubly excited determinants, this requires computation of the determinant of the overlap matrix between the two basis sets (with rows and columns rearranged to correspond to each double excitation). Given that there are  doubly excited determinants each for the bra and the ket (where *n*_o_ and *n*_v_ are the number of occupied and virtual MOs, respectively), and that each matrix determinant requires  computational effort, the total cost of a single AAT element at the MP2 level using numerical differentiation requires , in addition to the need for complex arithmetic.

By contrast, the analytic-derivative approach requires evaluation of eq 31, which scales at most as  and avoids complex wave function representations. While our implementation is not yet optimal, the computation of the entire MP2 AAT for (*P*)-hydrogen peroxide with the 6–31G basis set required only a few seconds using the analytic-derivative approach and all electrons active, whereas the numerical differentiation required several hours for each tensor element, even with the oxygen 1 s core orbitals frozen. The new analytic-gradient formulation makes possible MP2VCD simulations for larger molecules and basis sets, such as those below for (*S*)-methyloxirane which are impractical using the numerical approach.

### 4.2 MP2VCD Spectrum of (*S*)-Methyloxirane

**Figure 1:**
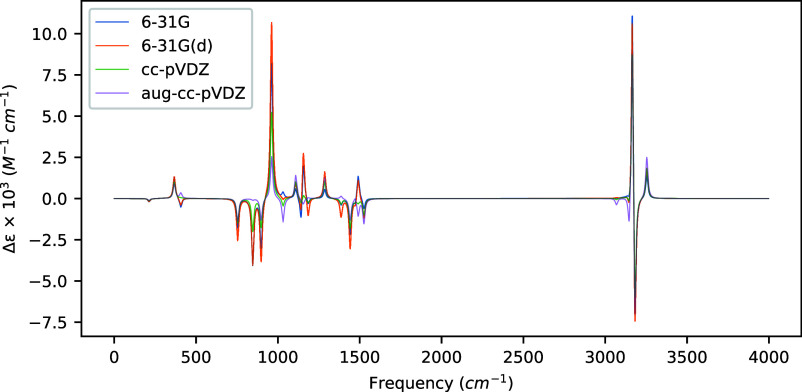
VCD spectra of (*S*)-methyloxirane computed at the MP2 level of theory for several basis sets using a common optimized geometry and Hessian obtained at the MP2/aug-cc-pVDZ level of theory.

**Figure 2:**
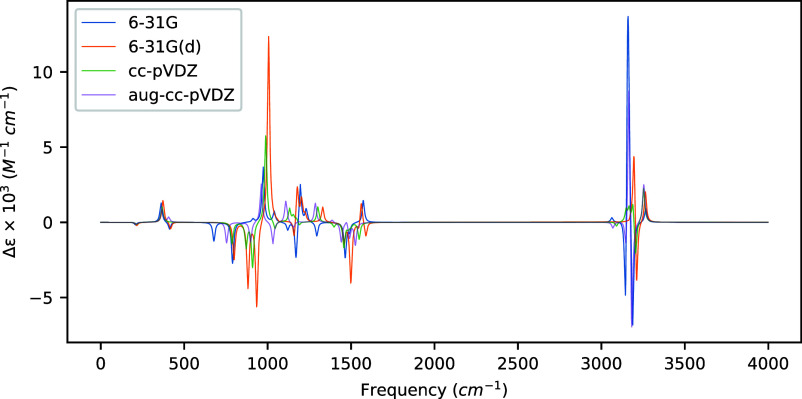
VCD spectra of (*S*)-methyloxirane computed at the MP2 level of theory for several basis sets using optimized geometries and Hessians computed at the same level as the rotatory strengths.

Using this new formulation of the AATs, we have computed MP2-level VCD rotatory strengths for (*S*)-methyloxirane for the 6–31G, 6–31G(d), cc-pVDZ, and aug-cc-pVDZ basis sets using two different approaches. Harmonic vibrational frequencies and rotatory strengths are reported for (*S*)-methyloxirane in Table 2 with a common geometry and common Hessian computed at the MP2/aug-cc-pVDZ level of theory. In addition, the corresponding VCD spectra are presented in Figure 1. Similarly, vibrational frequencies and rotatory strengths computed using geometries and Hessians computed at the same level as the rotatory strengths are reported in Table 3 with the spectra displayed in Figure 2. The choice of these modest basis sets helps to elucidate the variation in the MP2 rotatory strengths upon the addition of polarization functions on carbon and oxygen, polarization functions on hydrogen, and diffuse functions. In addition, the choice of using a common geometry/Hessian vs separate geometries/Hessians allows us to separate structural vs electronic effects in the rotatory strengths.

**Table 2:** VCD Rotatory Strengths of (*S*)-methyloxirane Computed at the MP2 Level of Theory for Several Basis Sets Using a Common Optimized Geometry and Hessian Obtained at the MP2/aug-cc-pVDZ Level

frequency (cm^–1^)	rotatory strength (10^–44^ esu^2^ cm^2^)			
	6–31G	6–31G(d)	cc-pVDZ	aug-cc-pVDZ
3254	2.495	3.302	3.281	4.506
3181	–17.108	–17.513	–14.127	–15.884
3164	37.584	33.418	27.892	25.338
3160	–17.312	–12.981	–11.250	–7.184
3147	–0.911	–1.928	–1.444	–4.229
3068	–0.024	0.090	–0.167	–0.724
1526	–2.477	–4.611	–3.705	–5.622
1490	5.876	5.177	–0.835	–3.953
1473	–1.502	–1.705	–0.408	1.037
1442	–8.791	–12.149	–7.154	–5.205
1386	–1.543	–4.539	–1.347	0.702
1286	2.527	7.301	4.633	5.736
1185	–2.145	–6.069	–1.366	0.277
1156	12.106	15.764	1.350	–1.706
1142	–9.001	–7.326	–2.005	–0.683
1109	3.177	4.890	5.447	7.494
1031	1.739	–1.030	–2.964	–8.265
961	49.773	64.918	31.811	15.573
898	–19.628	–25.045	–11.611	–8.674
846	–27.450	–27.241	–13.475	–0.573
754	–13.513	–19.358	–11.231	–10.496
406	–8.237	–6.523	0.230	4.505
367	21.043	21.287	15.800	14.207
212	–5.346	–5.160	–3.644	–3.375

**Table 3:** VCD Rotatory Strengths of (*S*)-methyloxirane Computed at the MP2 Level of Theory for Several Basis Sets Using Optimized Geometries and Hessians Computed at the Same Level as the Rotatory Strengths^a^

6–31G		6–31G(d)		cc-pVDZ		aug-cc-pVDZ	
freq	rot. str.	freq	rot. str.	freq	rot. str.	freq	rot. str.
3265	1.636	3263	3.672	3253	4.073	3254	4.506
3187	–14.649	3210	–9.250	3204	–4.480	3181	–15.884
3163	15.085	3196	9.875	3189	2.766	3164	25.338
3156	19.904	3180	0.509	3165	1.637	3160	–7.184
3145	–17.195	3167	–0.217	3148	1.437	3147	–4.229
3063	0.557	3108	–0.010	3091	–0.508	3068	–0.724
1573	6.154	1588	–3.549	1548	–4.130	1526	–5.622
1573	–0.692	1561	5.633	1498	–0.985	1490	–3.953
1557	–0.203	1546	–2.039	1483	–1.420	1473	1.037
1490	–2.111	1498	–15.452	1457	–6.593	1442	–5.205
1465	–9.245	1458	–5.317	1397	–1.216	1386	0.702
1294	–4.141	1330	4.396	1301	4.587	1286	5.736
1231	3.987	1225	2.176	1190	–1.165	1185	0.277
1195	13.263	1203	7.003	1172	1.077	1156	–1.706
1170	–12.766	1178	11.823	1154	2.005	1142	–0.683
1120	–2.652	1159	–6.617	1131	4.624	1109	7.494
1039	3.918	1070	–0.714	1044	–3.006	1031	–8.265
973	22.078	1005	71.797	988	33.915	961	15.573
912	1.363	934	–35.039	910	–19.030	898	–8.674
790	–19.944	882	–28.207	871	–11.066	846	–0.573
678	–10.592	799	–17.916	787	–10.455	754	–10.496
412	–6.849	418	–6.702	409	–0.073	406	4.505
361	21.032	372	22.692	367	16.270	367	14.207
208	–4.897	216	–5.916	214	–3.874	212	–3.375

a The units of frequency and rotatory strengths are cm^–1^ and 10^–44^ esu^2^ cm^2^, respectively.

We observe in Table 2 that, even using a common geometry and Hessian, there is considerable basis-set dependence in the rotatory strengths, including multiple instances of sign changes relative to the aug-cc-pVDZ basis set results. For example, for the normal modes at 1156 cm^–1^ (hydrogen rocking vibration), 1185 cm^–1^ (also a hydrogen rocking vibration), and 1386 cm^–1^ (an in-phase bending vibration primarily associated with the hydrogens on the methyl group), the three smallest basis sets yield the opposite sign rotatory strengths from the aug-cc-pVDZ basis set. These same sign differences, though weak, can be seen in the VCD spectrum in Figure 1. For the stronger rotatory strengths, we observe no sign changes across the basis sets, though the significance of diffuse functions can still be seen in the mode at 846 cm^–1^ (a ring breathing vibration), where the aug-cc-pVDZ basis set yields a rotatory strength factor of 24–48 times smaller than the other sets. Interestingly, the five highest frequency modes (all C–H stretching motions) are relatively well described by all the basis sets considered here.

When we use geometries and Hessians computed at the same level of theory as the rotatory strengths, as shown in Table 3 and Figure 2, we observe much greater variation in the resulting spectra, even in the high-frequency regime. For example, the C–H stretching vibrations at 3160 and 3147 cm^–1^ with the aug-cc-pVDZ basis set exhibit a sign change compared to the cc-pVDZ basis set (and compared to all three smaller basis sets for the former). Indeed, we observe sign reversals between cc-pVDZ and aug-cc-pVDZ for eight vibrational modes, though primarily for relatively weak rotatory strengths. Even for modes for which the signs are consistent, however, we observe large changes in the magnitude of the rotatory strength among the basis sets. For example, for the five modes with an absolute rotatory strength greater than 10.0 × 10^–44^ esu^2^ cm^2^ at the MP2/aug-cc-pVDZ level, three exhibit an intensity shift larger than a factor of 2 between MP2/cc-pVDZ and MP2/aug-cc-pVDZ.

## 5 Conclusions

In this work, we have derived and implemented an analytic-gradient method for computing VCD AATs at the MP2 level of theory. We have compared this method with that of our recently developed numerical-differentiation procedure—which is several orders of magnitude more computationally expensive and less precise than our new formulation—and obtained very good agreement between the two approaches. Using this new implementation, we also report the first MP2 level VCD spectra of (*S*)-methyloxirane using several modest basis sets.^34^

It remains to be seen whether the inclusion of electron correlation effects will be sufficient to resolve problematic cases, such as that of induced chirality observed in the CDCl_3_ solvent in the VCD spectrum of pugleone,^35^ or whether other important factors such as solvent models or anharmonicity must also be included. Nevertheless, this work represents a key step toward the simulation of fully analytic MP2-level VCD spectra for a wide array of molecules and basis sets, and it opens the door to future implementations of VCD at even higher levels of theory. Although the present effort does not include the use of GIAOs, resulting in origin-dependent rotatory strengths, our future work will be directed toward the development of a gauge- and origin-invariant formulation, as well as subsequent benchmark studies of other effects such as basis-set completeness.

## Supporting Information Available

Atomic coordinates of the test molecules (PDF)
