# Supplementary material for: Analytic Computation of Vibrational Circular Dichroism Spectra Using Second-Order Møller–Plesset Perturbation Theory
Source: J Chem Theory Comput. 2025 Mar 25;21(7):3504–12. doi: 10.1021/acs.jctc.5c00047 (PMC11983703; doi:10.1021/acs.jctc.5c00047)
Supplement: Supplementary file 1 — ct5c00047_si_001.pdf [file ct5c00047_si_001.pdf]

Analytic Computation of Vibrational Circular Dichroism Spectra Using Second-Order Møller-Plesset Perturbation Theory

Brendan M. Shumberger, Kirk C. Pearce, and T. Daniel Crawford*

Department of Chemistry, Virginia Tech, Blacksburg, Virginia, U.S.A.

E-mail: crawdad@vt.edu

Table S1: (*P*)-hydrogen peroxide MP2/cc-pVDZ optimized geometry (a.u.)

Atom Type	Atom Number	X	Y	Z
H	1	-1.780954530308296	1.411647335546379	0.872055376436941
H	2	1.780954530308296	-1.411647335546379	0.872055376436941
O	3	-1.371214332646589	-0.115525249760340	-0.054947416764017
O	4	1.371214332646589	0.115525249760340	-0.054947416764017

Table S2: (*S*)-methyloxirane MP2/6-31G optimized geometry (a.u.)

Atom Type	Atom Number	X	Y	Z
C	1	-0.436411366273077	0.151534026136331	-0.939761163240479
O	2	1.640929779484701	-1.475955748112904	0.229775802597979
C	3	1.773370434675130	1.409936624603667	0.251697621431119
H	4	3.266332758107407	2.203963624362215	-0.916860641738861
H	5	1.563779218351836	2.248453838254109	2.118642754350623
C	6	-2.978972409740116	0.029554840014890	0.358219721642582
H	7	-4.175591788570781	1.623871670248584	-0.229269844452829
H	8	-3.959968806575508	-1.736982731948496	-0.110722916700695
H	9	-2.722998791137711	0.108339816977296	2.415644537136533
H	10	-0.463127430470562	0.032774631807676	-2.996748764110896

Table S3: (S)-methyloxirane MP2/6-31G(d) optimized geometry (a.u.)

Atom Type	Atom Number	X	Y	Z
C	1	-0.400247379252466	0.101479377827636	-0.926326196754800
O	2	1.538674693348130	-1.400677899209889	0.258601292016535
C	3	1.807520340148573	1.309675044540570	0.226064950609519
H	4	3.328413207126016	2.025017045959494	-0.958331957804974
H	5	1.608376482966247	2.226537609298951	2.056558608656557
C	6	-2.936022197320953	0.085361691815703	0.344803643981329
H	7	-4.111426855204313	1.641661720201308	-0.343350645137738
H	8	-3.923439971527713	-1.690030321270871	-0.032924217347689
H	9	-2.700841855175627	0.275965778217362	2.387057878162288
H	10	-0.418409719756681	-0.068138117329203	-2.980827062932704

Table S4: (S)-methyloxirane MP2/cc-pVDZ optimized geometry (a.u.)

Atom Type	Atom Number	X	Y	Z
C	1	-0.400208448884393	0.083346078878714	-0.931435478741626
O	2	1.535666546265060	-1.394771029507393	0.269403769739990
C	3	1.819142279337406	1.307113638113703	0.220056147004876
H	4	3.346562491669792	2.016038830326753	-0.984863029121345
H	5	1.604313450908801	2.251303219675088	2.051347308139407
C	6	-2.941811007157573	0.095082612007084	0.345171618695598
H	7	-4.103993291961499	1.681315159914367	-0.340825099532328
H	8	-3.963684530966615	-1.676030627460780	-0.044832368509016
H	9	-2.705572018314348	0.270815496500312	2.403619978875454
H	10	-0.417130944755564	-0.095511692533100	-2.999707618332652

Table S5: (S)-methyloxirane MP2/aug-cc-pVDZ optimized geometry (a.u.)

Atom Type	Atom Number	X	Y	Z
C	1	-0.406643263371948	0.103169698978202	-0.932904009322372
O	2	1.550888890337005	-1.412171718063139	0.260072952566956
C	3	1.810064371203058	1.327408273345544	0.226984115881429
H	4	3.344838669765579	2.027224623297944	-0.964879943170253
H	5	1.599930911340288	2.226009101570543	2.076678955772736
C	6	-2.946240158335548	0.082079933386530	0.348469914386564
H	7	-4.122138549897469	1.649720715533630	-0.352252535246005
H	8	-3.940556043431783	-1.702386417970233	-0.037902074947365
H	9	-2.712874671266827	0.277413155894713	2.403143387960141
H	10	-0.412850332112492	-0.076749763394509	-2.996238617375079
